# Mayan Medicinal Plants *Bignonia potosina* and *Thouinia paucidentata* Demonstrate Anti-Infective Properties Against the Priority Antibiotic-Resistant Bacteria *Acinetobacter baumannii* and *Pseudomonas aeruginosa*

**DOI:** 10.3390/plants13243498

**Published:** 2024-12-14

**Authors:** Gloria María Molina-Salinas, Angel Dzul-Beh, Andrés Humberto Uc-Cachón, Haziel Eleazar Dzib-Baak, Avel Adolfo González-Sánchez, Geovani Antonio Palma-Pech, Carlos Javier Quintal-Novelo

**Affiliations:** 1 Unidad de Investigación Médica Yucatán, Instituto Mexicano del Seguro Social, Mérida 97150, Yucatán, Mexico; angeldzulbeh1992@gmail.com (A.D.-B.); andresuccachon@gmail.com (A.H.U.-C.); hazieldzibbaak@gmail.com (H.E.D.-B.); 2 Facultad de Ingeniería Química, Universidad Autónoma de Yucatán, Mérida 97150, Yucatán, Mexico; avel.gonzalez@correo.uady.mx; 3 Campus de Ciencias Biológicas y Agropecuarias, Universidad Autónoma de Yucatán, Mérida 97100, Yucatán, Mexico; geovani.palma@correo.uady.mx; 4 Unidad Médica de Alta Especialidad, Centro Médico Ignacio García Téllez, Instituto Mexicano del Seguro Social, Mérida 97150, Yucatán, Mexico; quintal99@gmail.com

**Keywords:** *Acinetobacter baumannii*, antibacterial, anti-resistance, anti-virulence, *Bignonia potosina*, efflux pump, *Pseudomonas aeruginosa*, pyocyanin, *Thouinia paucidentata*

## Abstract

(1) Background: Carbapenem-resistant *Acinetobacter baumannii* (CBRAB) and *Pseudomonas aeruginosa* (CBRPA) are critical and high-priority pathogens that require new therapeutic developments. Medicinal plants are valuable pharmaceutical resources. This study explored the anti-infective properties of Mayan plants, *Bignonia potosina*, and *Thouinia paucidentata*. (2) Methods: Plant parts were extracted using *n*-hexane, and their ability to inhibit bacterial growth and counteract resistance mechanisms and virulence factors in CBRAB and CBRPA was assessed. GC-MS analysis of the composition of the non-polar extracts and chemometric techniques correlated the phytoconstituents with anti-infective properties. (3) Results: *Bignonia potosina* liana and flower extracts exhibited potent antibacterial activity against *A. baumannii* strains (MIC 15.7 to 250 µg/mL) and moderate activity against *P. aeruginosa* strains (MIC 250 to 1000 µg/mL). *Thouinia paucidentata* leaf extract at 1000 µg/mL reduced imipenem MIC by 2048-fold for CBRAB, and *B. potosina* flower extract significantly inhibited *A. baumannii* catalase activity (at 62.5 µg/mL) and reduced *P. aeruginosa* pyocyanin production (at 1000 µg/mL). Chemometric analysis identified fatty acids, fatty acid amides, terpenes, and higher alkanes as contributors to their anti-infective properties. (4) Conclusions: This study highlights the potential of medicinal plants in the development of novel anti-infective therapies against CBRAB and CBRPA with various targets.

## 1. Introduction

Antimicrobial resistance (AMR) is a significant threat to public health in the 21st century, as evidenced by the increasing trend of treatment failure with medications commonly used to treat infectious diseases caused by parasites, fungi, bacteria, and viruses. Although antibiotic therapy is widely regarded as one of the most significant advancements in medical history [1], the widespread and inappropriate use of these drugs among outpatients has resulted in drug resistance, thereby ending the golden era of antibiotic discovery [2]. The World Health Organization (WHO) has listed priority bacterial pathogens to combat AMR, calling for the development of new anti-infective agents and public health interventions. Carbapenem-resistant *Acinetobacter baumannii* (CBRAB), a member of the critical-priority group and, *Pseudomonas aeruginosa* (CBRPA), a member of the high-priority group, are the most common bacteria that cause both healthcare and community infections [3]. These pathogens pose significant epidemiological threats, emphasizing the urgent need for immediate action to curb their spread and protect public health [1,4].

Medicinal flora is a rich source of phytochemical compounds with pharmacological properties that offer an outstanding reservoir for the development of new drugs [5]. Currently, the pharmacological effects of medicinal plants are recognized as a promising approach for future drug development, particularly considering the emergence of superbugs [6]. Mayan medicine is a traditional healthcare system that has been utilized in regions such as Guatemala, Mexico (including the Yucatan Peninsula, Chiapas, and Tabasco), Belize, western Honduras, and El Salvador. This system has cultural significance in Mexico and continues to be used today, including the use of medicinal flora for the treatment of illnesses and promotion of overall health [7]. Studies on Mayan medicinal plants have shown a wide range of biological activities, including antidiabetic, anticancer, antioxidant, and antimicrobial effects, as well as various structural chemical metabolites [8,9,10].

*Bignonia potosina* (K. Schum. & Loes.) L.G. Lohmann, also known as *Cydista potosina,* is a liana species that belongs to the Bignoniaceae family. This genus includes six lowland species ranging from central and southern Mexico to Paraguay and eastern Brazil. The Mayan name is éek’ k’iix il and the common name is ‘’hierba trepadora”. It is used medicinally for coughs, colds, wound healing, wasps and scorpion stings, as well as bile and kidney diseases [11,12,13]. Its activity against methicillin-resistant *Staphylococcus aureus* (MRSA) biofilms has recently been demonstrated [9]. *Thouinia paucidentata* Radlk. is a timber tree from the tropical forests of the Yucatan Peninsula that belongs to the Sapindaceae family. The Mayan name is k’an chuunup, which is used to treat skin infections, sores, coughs, and diarrhea [14,15]. Our research group has identified the anti-growth and anti-biofilm activities of *T. paucidentata* against MRSA [9], and recently reported its activity against the promastigote and amastigote forms of *Leishmania mexicana*, germacrene D-4-ol, thunbergen, and thunbergol, the most abundant phytoconstituents in the bioactive leaf extract [16]. Our team focused on exploring the therapeutic potential of Mayan flora as an anti-infective agent against WHO bacterial priority pathogens. This includes targeting bacterial growth, resistance mechanisms, and virulence factors to neutralize pathogens. In this study, we analyzed the properties of *B. potosina* and *T. paucidentata* non-polar extracts using these three approaches against CBRAB and CBRPA. Additionally, we employed gas chromatography–mass spectrometry (GC-MS) to examine the chemical composition of extracts and utilized chemometric techniques to identify correlations between phytoconstituents and anti-infective properties.

## 2. Results

### 2.1. Chemical Composition

GC–MS was used to identify 22 compounds in the *n*-hexane (*n*-Hex) extracts of *B. potosina*. Fatty compounds, particularly hexadecanamide and hexadecanoic acid, were the predominant phytoconstituents in the liana and flower *n*-Hex extracts, respectively. In contrast, squalene was the most abundant compound in the leaf extract accounting for 42.8% of the total peak area. Hexadecanamide, octadecanamide, tetratriacontane, and hexatriacontane were present in all three extracts, albeit in variable proportions (Table 1).

In the *n*-Hex extracts of *T. paucidentata*, the chemical composition analyzed by GC-MS varied considerably between bark and leaf. Oxygenated terpenes such as caryophyllene oxide and kolavelool were identified as the major compounds in the *n*-Hex bark extract. Conversely, thunbergen, cubebol, and oplopanone were the predominant compounds in the *n*-Hex leaf extract, accounting for 50% of the total chromatogram area. Other abundant compounds identified exclusively in the *n*-Hex leaf extract included cembrenol, β-caryophyllene, α-tocopherol, and β-copaene (Table 2).

### 2.2. Antibacterial Activity

Table 3 and Table 4 show the antibacterial activities of the plant *n*-Hex extracts *against A. baumannii* and *P. aeruginosa* strains. The liana extract of *B. potosina* exhibited strong inhibition against *A. baumannii* strains, with MIC (minimum inhibitory concentration) values ranging from 15.7 to 31.25 µg/mL, but showed low activity against *P. aeruginosa*, with MIC values between 250 and 1000 µg/mL. Furthermore, the *B. potosina* flower extract demonstrated moderate inhibition of *A. baumannii* strains (MIC = 62.5–250 µg/mL) and low activity against one *P. aeruginosa* strain (PAE-UIMY-79, MIC = 1000 µg/mL). Moreover, extracts obtained from the leaf of *B. potosina* and bark and leaf of *T. paucidentata* were effective against specific strains of *A. baumannii*.

### 2.3. Biofilm Formation Inhibition

**Figure 1:**
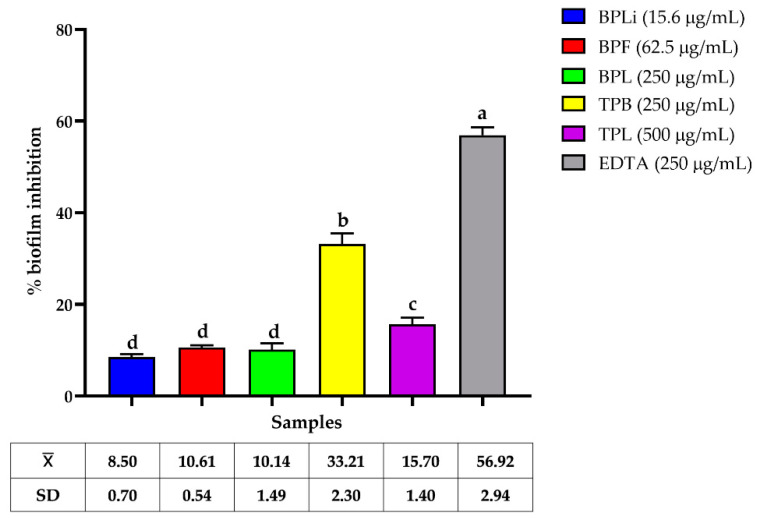
Anti-biofilm activity of *Bignonia potosina* and *Thouinia paucidentata n*-Hex extracts against strong biofilm-producing CBRAB (UIMY-ABA-81). Values ± SD with the same letter showed non-significant differences in the post hoc Tukey test (*p* < 0.05). From *B. potosina*: BPLi: *n*-Hex liana extract; BPF: *n*-Hex flower extract; BPL: *n*-Hex leaf extract. From *T. paucidentata* TPB: *n*-Hex bark extract; TPL: *n*-Hex leaf extract.

**Figure 2:**
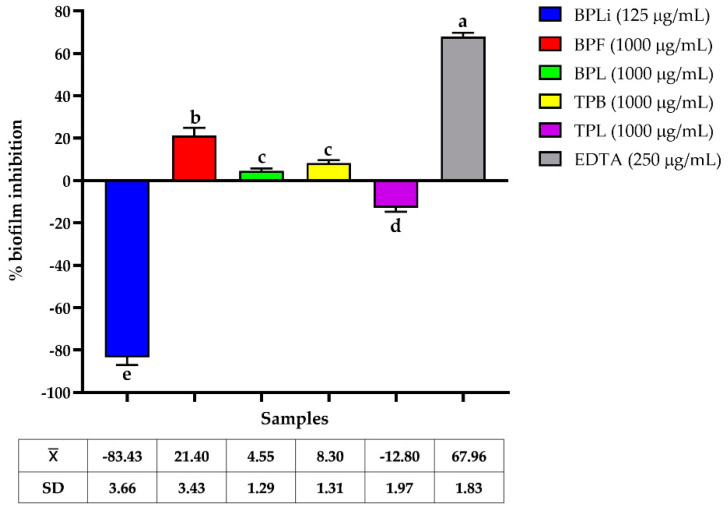
Anti-biofilm activity of *Bignonia potosina* and *Thouinia paucidentata* extracts against strong biofilm-producing CBRPA (*Pseudomonas aeruginosa* ATCC-35032) at sub-inhibitory concentrations. Values ± SD with the same letter showed non-significant differences in the post hoc Tukey test (*p* < 0.05). From *B. potosina*: BPLi: *n*-Hex liana extract; BPF: *n*-Hex flower extract; BPL: *n*-Hex leaf extract. From *T. paucidentata* TPB: *n*-Hex bark extract; TPL: *n*-Hex leaf extract.

All plant *n*-Hex extracts exhibited low biofilm inhibition of the CBRAB (UIMY-ABA-81) and CBRPA (ATCC-35032) strains, with some enhancing biofilm production (Figure 1 and Figure 2). The bark *n*-Hex extract of *T. paucidentata* demonstrated the highest biofilm inhibition (33.21 ± 2.3%) at 250 µg/mL (MIC/2) against CBRAB (UIMY-ABA-81) strain (Figure 1).

### 2.4. Antibiotic-Modulation Activity

In our study, we also investigated the antibiotic-modulating activity of the *n*-Hex plant extracts of *B. potosina* and *T. paucidentata* against clinical isolates of CBRAB and CBRPA (Table 5 and Table 6). Surprisingly, *T. paucidentata* leaf extract (1000 µg/mL) led to a 2048-fold reduction in the MIC values of imipenem (IMP), followed by *B. potosina* leaf extract (BPL^a^: 1000 µg/mL) with an antibiotic-modulating factor (AMF) value of 16 (Table 5). Moreover, all extracts from both plants showed low meropenem (MEM)-modulating activity (2-to 4-fold) against *P. aeruginosa* (Table 6).

### 2.5. Inhibition of Catalase Activity

**Figure 3:**
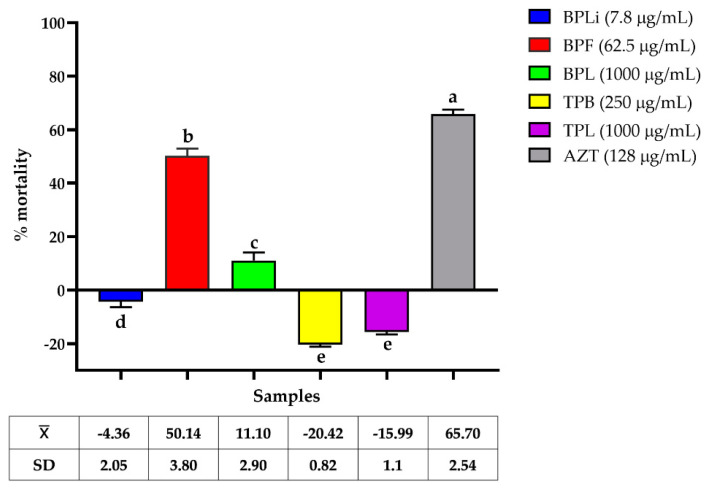
Effect of *Bignonia potosina* and *Thouinia paucidentata* extracts on CBRAB UIMY-ABA-205 after treatment with H_2_O_2_. Values ± SD with the same letter showed non-significant differences in the post hoc Tukey test (*p* < 0.05). From *B. potosina*: BPLi: *n*-Hex liana extract; BPF: *n*-Hex flower extract; BPL: *n*-Hex leaf extract. From *T. paucidentata* TPB: *n*-Hex bark extract; TPL: *n*-Hex leaf extract; AZT: azithromycin.

CBRAB UIMY-ABA-205 was pretreated with MIC/2 of plant extracts, after which the bacteria were exposed to reactive oxygen species (ROS) to assess their survival capacity. As illustrated in Figure 3, only the *B. potosina* flower extract significantly reduced the survival of UIMY-ABA-205 to ROS, resulting in 49.14% ± 3.80 mortality.

### 2.6. Inhibition of Pyocyanin Production

**Figure 4:**
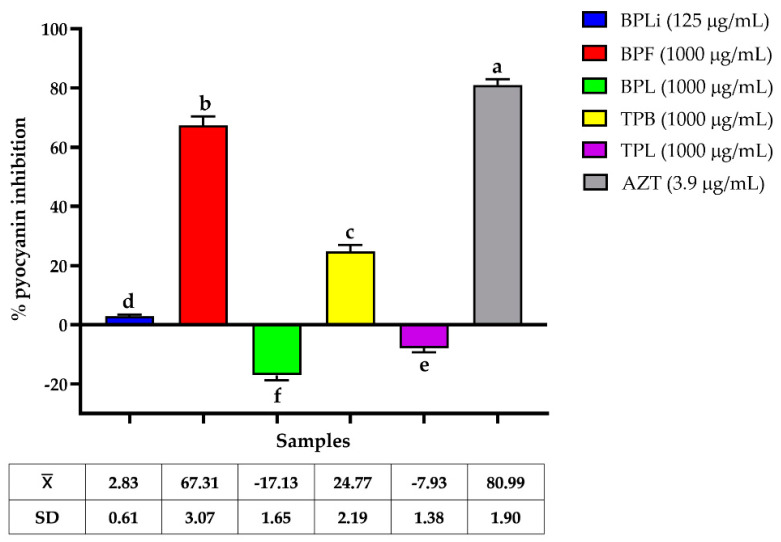
Effect of *Bignonia potosina* and *Thouinia paucidentata* extracts on the production of pyocyanin of CBRPA PAE-ATCC-35032. Values ± SD with the same letter showed non-significant differences in the post hoc Tukey test (*p* < 0.05). From *B. potosina*: BPLi: *n*-Hex liana extract; BPF: *n*-Hex flower extract; BPL: *n*-Hex leaf extract. From *T. paucidentata* TPB: *n*-Hex bark extract; TPL: *n*-Hex leaf extract; AZT: azithromycin.

The effects of the *n*-Hex plant extract on pyocyanin production by CBRPA PAE-ATCC-35032 were evaluated. As illustrated in Figure 4, the flower extract from *B. potosina* reduced pyocyanin by 67.31 ± 3.07% when tested at 1000 µg/mL, followed by the bark extract of *T. paucidentata* at 1000 µg/mL.

### 2.7. Chemometric Analysis-Based GC-MS Analysis

**Figure 5:**
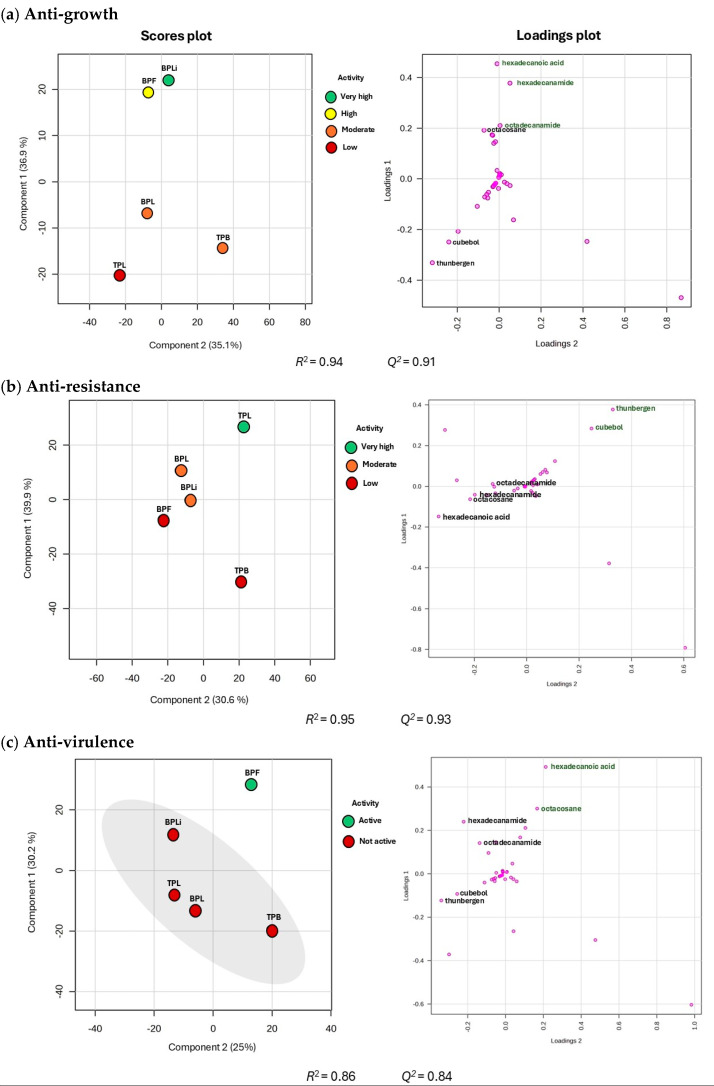
(**a**) Scores and loading plots from the PLS-DA model for activity on bacterial growth. MIC values of *n*-Hex extracts from *Bignonia potosina* and *Thouinia paucidentata* against CBRAB and CBRPA strains according to this categorical classification: 1000 µg/mL: low activity; 500–250 µg/mL: moderate activity; 125–62.5 µg/mL: high activity; <62.5 µg/mL: very high activity. (**b**) Scores and loadings plot from the PLS-DA model for antibiotic-modulation activity according to this categorical classification: 2–4 of AMF: low activity; 8–16 of FMA: moderate activity; >128 of FMA very high activity. (**c**) Scores and loadings plot from PLS-DA model for antivirulence activity according to this categorical classification: % inhibition > 50%: active. Phytoconstituents in green are associated with anti-infective properties. BPLi: *n*-Hex liana extract of *B. potosina*; BPF: *n*-Hex flower extract **B. potosina**; BPL: *n*-Hex leaf extract *B. potosina*; TPB: *n*-Hex bark extract of *T. paucidentata*; TPL: *n*-Hex leaf extract of *T. paucidentata*.

To identify compounds associated with anti-infective activity (antibacterial, anti-resistance, and anti-virulence effects) against CBRAB and CBRPA strains, a Partial Least Squares Discriminant Analysis (PLS-DA) of the GC-MS data obtained from the *n*-Hex extracts of *B. potosina* and *T. paucidentata* was conducted considering the extent of biological activity as Y variable. Based on our results, three PLS-DA models were constructed (Figure 5), and the putative biomarkers associated with anti-infective activity were identified, which were either exclusive to or were present at higher concentrations in the extracts that exhibited the highest activity (Table 7, Table S1).

For the antibacterial activity model, the PLS-DA score plot showed that the liana and flower *n*-Hex extracts from *B. potosina* had the highest activity. The model fitting ability (R^2^) was 0.94, whereas the predictive ability (Q^2^) was 0.91, with a cumulative variance of 72% (Figure 5a, Figure S1). The compounds responsible for projection in the score plot were identified by analyzing the loading plot and the variable importance in projection (VIP) value for each compound. Only compounds with VIP > 1 were considered significant for the discrimination of the most active groups. Consequently, three compounds influenced the discrimination of the high and very high activity groups in PLS-DA (Table 7): hexadecanamide (*r* = 0.36; VIP = 2.75), hexadecanoic acid (*r* = 0.45; VIP = 2.37), and octadecanamide. (*r* = 0.24 VIP = 1.47).

For the PLS-DA model antibiotic-modulation activity, the Q^2^ (0.95) and R^2^ (0.93) values indicated that the model was appropriate for the two components, with a cumulative variance of 70.5% (Figure 5b, Figure S2). The compounds responsible for the discrimination of the “very high-activity” group were thunbergen (*r* = 0.24; VIP= 1.47) and cubebol (*r* = 0.24; VIP = 1.47), which were the major compounds identified in the *n*-Hex leaf extract of *T. paucidentata* (Table 7).

Finally, the PLS-DA model for anti-virulence activities (Q^2^ = 0.86, R^2^ = 0.76) was associated with hexadecanoic acid (*r* = 0.48, VIP = 3.45), and octacosane (*r* = 0.30, VIP = 2.50) as potential contributors to the inhibition of catalase activity and pyocyanin production by *n*-Hex extract flower from *B. potosina* (Figure 5c, Table 7, Figure S3).

### 2.8. Cytotoxicity

The cytotoxicity of the *n*-Hex plant extracts against Vero cells is shown in Table 8. The *n*-Hex extract of *B. potosina* liana displayed the most significant cytotoxic effect, with a cytotoxic concentration of 90% (CC_90_) of 235.2 ± 4.5. Despite its high cytotoxicity, this extract showed the best selectivity index (SI) against *A. baumannii* strains, with values between 15.0 and 7.5.

## 3. Discussion

AMR is a primary public health threat. The WHO and others, such as the Centers for Disease Control and Prevention in the USA and Europe, promote the research and development of novel antibiotics, particularly for critical and high-priority pathogens, such as CBRAB and CBRPA [3,17,18]. Plants are a significant source of new antibiotics [19]. In this study, we investigated the antibacterial, anti-resistance, and anti-virulence properties of two Mayan medicinal plants against CBRAB and CBRPA.

In this study, we analyzed the chemical composition of the *n*-Hex extracts of *B. potosina* and *T. paucidentata*, which are crucial for explaining and supporting the anti-infective activity of medicinal plants. To the best of our knowledge, there have been no previous phytochemical studies of *B. potosina*. The chemical composition of the *n*-Hex extracts from this plant revealed the predominant presence of fatty acids and linear alkanes, except for the leaf extract, which contained a high percentage of squalene. Phytochemical studies on plants of the genus *Bignonia* have characterized fatty acids such as hexadecanoic, stearic, linoleic, and linolenic acids, as the main phytoconstituents of hexane extracts [11,20]. In another study, the chemical profile of the *n*-Hex extract of *Bignonia africana* was characterized by the presence of cetylic, elaidic, stearic, and linoleic acids, and methyl palmitate. Our group previously reported the chemical composition of *n*-Hex *T. paucidentata* leaf extract, with sesquiterpenes and diterpenes, including thunbergen, as the predominant [16]. These metabolites potentially contribute to biological activity, emphasizing the importance of analyzing plant extracts to identify the key bioactive compounds.

Regarding biological activity, the *n*-Hex extracts from *B. potosina* liana demonstrated greater growth inhibition against both pathogens, exhibiting strong efficacy against *A. baumannii* strains and mild effectiveness against *P. aeruginosa*. This is the first report of the anti-CBRAB, and anti-CBRPA properties of *B. potosina* extracts. Our previous study on *B. potosina* extracts against *S. aureus* yielded no anti-staphylococcal extracts [9]. To date, no studies have explored the antimicrobial activity of species of the genus *Bignonia*.

Chemometric analysis using GC-MS revealed that the potent activity of *the B. potosina* liana extract was associated with the concentrations of the fatty acid amides hexadecanamide, octadecanamide, and hexadecanoic acid. Although these fatty acid amides lack previous reports against both bacteria, these classes of compounds have been associated with antibiotic properties owing to the inhibition of bacterial cellular lipid biosynthesis [21]. Hexadecanoic acid showed moderate activity at 50 µg/mL against *S. aureus*, *Bacillus subtilis*, *Escherichia coli*, and *Klebsiella pneumoniae* [22]. In liposomal formulations, it displayed MIC values of 0.5 μg/mL and 2 μg/mL against MDR *S. epidermidis* (ATCC 700566) and vancomycin resistant *Enterococcus faecalis* (VRE, ATCC 700802), respectively [23]. Various bacterial processes, including DNA and RNA synthesis, cell wall biosynthesis, protein synthesis, and metabolic pathways, are affected by fatty acids. Consequently, they are promising candidates for the development of next-generation antibacterial agents to treat a wide range of bacterial infections [24,25].

In contrast, *T. paucidentata* bark *n*-Hex extracts showed moderate to low antibacterial activity against CBRAB strains. Caryophyllene oxide was the majority compound identified in this extract and may contribute to the observed activity. A previous study demonstrated the antibacterial activity of caryophyllene oxide against *S. aureus* (ATCC6538P), *E. coli* (ATCC 8739), *K. pneumoniae* (clinical isolate), and *Salmonella abony* (DSM 4224), with MIC values of 60 µg/mL for all strains. The same study found that caryophyllene oxide was ineffective against *P. aeruginosa* (G28), which is consistent with our results showing the inactivity of *n*-Hex bark extract against *P. aeruginosa* [26].

AMR has increased significantly in recent years, resulting in a reduction in the efficacy of antibiotics that are considered a “last-resort” defense against MDR bacteria. Investigating the mechanisms of resistance inhibitors is the most promising strategy for combating AMR. Biofilms and efflux pumps are the primary mechanisms underlying antibiotic resistance in both Gram-positive and Gram-negative bacteria [27,28]. In this study, we evaluated the effects of plant extracts on carbapenem-resistant *A. baumannii* and *P. aeruginosa* biofilms formation and evaluated their carbapenem-modulation activity. The plant extracts exhibited low anti-biofilm formation activity against both pathogens, with the *n*-Hex extract from *T. paucidentata* bark displaying the most significant effect, reaching 33.21 ± 2.3% at 250 µg/mL (MIC/2). In a previous study, we reported that this extract demonstrated activity against *S. aureus* (ATCC-43300) biofilms with IC_50_ = 64.4 ± 5.5 µg/mL [9]. The predominant compounds identified were caryophyllene oxide (48%) and kolavelool (23.8%). Previous studies have reported the inhibition of *Candida albicans* biofilm formation by caryophyllene oxide alone [29].

Regarding carbapenem-modulation activity, we identified two clinical isolates in our biobank, one *A. baumannii* (UIMY-ABA-5) [30] and one *P. aeruginosa* (UIMY-PAE-167), which exhibited carbapenem resistance, that was suppressed by phenylalanine-arginine-β-naphthylamide, an efflux pump inhibitor. The *n*-Hex extract of *T. paucidentata* leaf demonstrated a potent inhibition of efflux pump, decreasing the MIC of IMP 2048-fold on CBRAB.

Chemometric analysis identified thunbergen and cubebol to be possibly responsible for the activity of this extract. There are no previous reports on the effects of these compounds on the efflux pump activity. Nevertheless, numerous terpenes are effective on efflux pumps, including those identified in the active extracts. For instance, α-humulene decreased the expression of bmeB1 and bmeB2 genes in *Bacteroides fragilis*, thereby reducing RND EP expression in this microorganism. In silico models revealed that α-amyrin has greater affinity for the binding sites of MepA and NorA efflux pumps. Moreover, studies have shown that caryophyllene and caryophyllene oxide effectively suppress the NorA, Tet(K), and MsrA efflux systems in *S. aureus* [31,32].

Currently, the development of pharmaceutical agents that inhibit virulence factors has garnered significant attention. This strategy seeks to neutralize pathogens by inhibiting bacterial virulence factors and disrupting pathogen–host interactions, thereby reducing host damage and disease severity. Furthermore, anti-virulence drugs may impose less selective pressure than antibiotics [33].

We examined the effects of plant extracts on the virulence factors of *A. baumannii* and *P. aeruginosa*. The flower *n*-Hex extract of *B. potosina* at MIC/2 (62.5 µg/mL) was effective in the H_2_O_2_ sensitivity assay, suggesting that it impedes the protective mechanisms of *A. baumannii* against ROS. This pathogen synthesizes enzymes, such as catalases and superoxide dismutase, to shield itself from reactive species, such as H_2_O_2_. It has been suggested that *Acinetobacter* species catalases may diminish reactive oxygen species production by phagocytic cells, enabling their survival [34]. Moreover, the flower *n*-Hex extract of *B. potosina* at MIC/2 (1000 µg/mL) suppressed pyocyanin production. This toxin damages host tissues, escalates disease severity, and compromises organ function [35].

GC-MS-based chemometric analysis revealed that the potent anti-virulence activity of *B. potosina n*-Hex flower extract against both CBRAB and CBRPA strains was associated with the alkane octacosane and hexadecenoic acid. No previous studies have reported on alkane activity against virulence factor production. Docking and molecular dynamics analyses revealed that hexadecanoic acid interacts with CviR, a transcriptional regulatory protein in the quorum-sensing system, implying that hexadecanoic acid inhibits this system [36]. The expression of catalase and superoxide dismutase in *A. baumannii* and pyocyanin in *P. aeruginosa* is regulated by a quorum-sensing system [37,38], suggesting that the *n*-Hex of *B. potosina* flowers may act through this pathway. Other studies on extracts rich in hexadecanoic acid have demonstrated activity against quorum-sensing system, and inhibition of *P. aeruginosa* [39,40]. *Spirulina platensis* extract (250 µg/mL), containing 47.68% hexadecanoic acid, inhibited violacein production by 90% in *Chromobacterium violaceum* ATCC-12472, and inhibited pyocyanin production by 75% in *P. aeruginosa* PA14 [39]. A recent study found that 100 µg/mL *Piper betel* leaf extract, which contains hexadecanoid acid (23.25%) as a major compound, drastically decreased quorum-sensing genes expression in *P. aeruginosa*, consequently diminishing pyocyanin production [40].

Multivariate analyses, such as PLS-DA, were employed to identify biomarkers associated with biological activity by extracting patterns from complex datasets and correlating the chemical composition with biological outcomes. Chemometric analysis identifies patterns in chemical matrices and the relationship between the composition and activity [41]. PLS-DA highlights the key compounds that contribute to biological effects, thereby facilitating biomarker discovery for therapeutic applications. Multivariate methods have successfully identified biomarkers of antimicrobial activity in plant extracts and essential oils [42,43] and have correlated metabolic profiles with anticancer effects [44], demonstrating their effectiveness in understanding plant extract composition and identifying potential biomarkers for phytodrug development.

Plant extracts were evaluated using a cytotoxicity model in Vero cells. The liana *n*-Hex extract from *B. potosina*, which exhibited the highest antibacterial activity against CBRAB, had an SI of 15.0. It is noteworthy that extracts with SI values greater than 10 are considered potential candidates for further investigation [45].

## 4. Materials and Methods

This project received approval from the Scientific and Ethics Committees National of the Instituto Mexicano del Seguro Social (IMSS), with approval numbers R-2019-785-089 and R-785-2023-014.

### 4.1. Medicinal Plant Extracts

#### 4.1.1. Collection

*Bignonia potosina* was collected in March 2019 from Maní Yucatán, Mexico (21°36′32.7″ N, 88°37′21.94″ W), and *Thouinia paucidentata* was collected in June 2020 from Xmatkuil Yucatán, Mexico (21°36′32.7″ N, 88°37′21.94″ W). Geovani Antonio Palma-Pech, a member of the Botany Department in the Faculty of Veterinary Medicine at the University Autonomous of Yucatan (UADY), authenticated the medicinal plants, and the specimens were subsequently deposited in the herbarium Alfredo Barrera Marín-UADY under voucher number 23,233 and 23,461, respectively. The Plant List was used to authenticate the botanical nomenclature and taxonomy of the medicinal herbs [46].

#### 4.1.2. Extracts Preparation

The parts of medicinal plants used in Mayan medicine were processed according to a standardized protocol [9]. Fresh bark, flower, leaf, and liana were cut, oven-dried at 40 °C for 72 h, and then ground. *n*-Hex extracts were obtained by soaking in an orbital shaker at 0.106× *g* for 24 h at room temperature. The solvent was evaporated under a vacuum using a rotary evaporator (Büchi, Flawil, Switzerland). Crude extracts were stored at −20 °C. in 100% dimethyl sulfoxide (DMSO; Sigma-Aldrich, St. Louis, MO, USA) [47].

#### 4.1.3. Chemical Analysis of Bioactive Extracts

*n*-Hex extracts were analyzed using a gas chromatograph (Trace 1310, Thermo Scientific; Waltham, MA, USA) coupled with a mass spectrometer (MS, ISQ LT, Thermo Scientific) and a TriPlus RSH autosampler. The extracts were diluted in HPLC-grade chloroform and ultra-high-purity helium was used as the carrier gas at a flow rate of 1 mL/min. Samples were injected in splitless mode with a 1.0 μL volume and 50 mL/min flow rate, using an HP-5MS UI capillary column as the stationary phase. The oven temperature was initially set at 50 °C for 1 min, raised to 220 °C (held for 2 min) at 4 °C min per min, and then to 300 °C (held for 1 min) at 5 °C min^−1^. The ionization energy was 70 eV, and mass spectra were obtained in the full scan mode from 50 to 700 amu. Compound identification was based on comparing the analyte mass spectra at specific retention times with the NIST05 MS Library standards and MS Search Program v.2.0. The area percentage of each component was calculated by comparing the average peak area with the total area, with a spectral matching factor cutoff of 700, excluding components below this threshold [48]

### 4.2. Anti-Infective Assays

#### 4.2.1. Pathogenic Bacteria

The ATCC strains and clinical isolates of *A. baumannii* and *P. aeruginosa* (Table 9 and Table 10) are part of the biobank of the Unidad de Investigación Médica Yucatán of IMSS and were maintained at −80 °C in tryptic soy broth (TSB; Becton Dickinson Co., Franklin Lakes, NJ, USA) supplemented with glycerol (J.T. Baker, Inc., Phillipsburg, NJ, USA).

#### 4.2.2. Activity of Plant Extracts on Bacterial Growth

The MIC of the *n*-Hex plant extracts against bacteria was determined using the resazurin microtiter assay (REMA) broth dilution method. Bacterial cultures grown on Muller–Hinton agar (MHA; Becton Dickinson Co.) were suspended in Muller–Hinton broth (MHB; Becton Dickinson Co.) to match the 0.5 McFarland standard turbidity. This suspension was diluted to 1:50 to prepare the working inoculum, and 100 μL of this suspension was incubated with 100 μL of MHB containing the plant extract at serial dilutions ranging from 1000 to 15.62 μg/mL. The assay included positive, negative, and sterility controls, and was conducted in duplicate three times independently. After a 16 h incubation at 37 °C, 30 μL of resazurin (Sigma-Aldrich) was added, and the plates were incubated for an additional 2 h at 37 °C. The pink color indicated bacterial growth, whereas the blue color indicated no growth. The MIC was defined as the highest dilution without a color change from blue to pink. The minimal bactericidal concentration (MBC) of each extract was determined by reseeding the bacterial culture. Five microliters of extract-treated bacterial suspensions at MIC, 2 × MIC, and ½ MIC in MHB, along with the controls, were transferred to a new microplate containing fresh medium. After incubation, resazurin was added. MBC was the minimal extract concentration that prevented a color shift in the reincubated cultures. The assay was performed in triplicate and the MBC/MIC index was calculated [48].

#### 4.2.3. Activity of Plant Extracts on Drug Resistance Mechanisms

##### Biofilm Formation Inhibition Assay

The crystal violet (CV) staining method in 96-well microplates was used to evaluate the biofilm bacterial inhibition of the CBRAB UIMY-ABA-81 and the CBRPA PAE ATCC35032, as previously described [30]. Strains grown on MHA were incubated in TSB at 37 °C for 24 h and then transferred to TSB containing 1% glucose (TSB + G) to achieve a McFarland standard turbidity of 0.5. The suspension was diluted 1:50, and 100 μL were mixed with 100 μL of MIC/2 of each *n*-Hex plant extract in TSB + G. Ethylenediaminetetraacetic acid (EDTA; Sigma-Aldrich) served as a positive control, and extract-free wells were used as negative controls. After 24 h of incubation at 37 °C, the wells were washed, dried, and stained with 0.1% CV (Sigma-Aldrich), and absorbance at 490 nm was measured to determine biofilm inhibition. All assays were conducted in triplicate, and the mean ± standard deviation was calculated. The percentage of inhibition of biofilm formation was determined.

##### Antibiotic-Modulating Activity Assay

The growth inhibitory effects of *n*-Hex plant extracts were investigated against the clinical isolates CBRAB UIMY-ABA-5 and CBRPA UIMY-PAE-167, which exhibited efflux pump-mediated resistance to IMP (Sigma-Aldrich) or MEM (Sigma-Aldrich), respectively. The assay was performed in 96-well microplates, with each well containing 50 μL of MHB and 50 μL of carbapenem solution (two-fold serial dilutions). Subsequently, 50 μL of extract at sub-inhibitory concentrations (MIC/2-MIC/8) were added, followed by 100 μL of bacterial inoculum (10^6^ CFU/mL). Controls included a positive control (cultures with efflux pump inhibitor phenylalanine-arginine-β-naphthylamide [(Sigma-Aldrich]), a negative control (culture-free wells of extracts), and a sterility control (culture broth alone). After incubation, MIC was determined using resazurin. The AMF was calculated as the MIC ratio of the antibiotic alone to the antibiotic plus extract to express the antibiotic-modulating effects of the extracts. Each assay was performed in duplicate [30].

#### 4.2.4. Activity of Plant Extracts on Virulence Factor

##### Inhibition of Catalase Activity Assay

To evaluate the effect of *n*-Hex plant extracts on the susceptibility of the CBRAB UIMY-ABA-205 to H_2_O_2_, bacterial cultures with subinhibitory growth concentrations (MIC/2) were incubated for 24 h at 36.5 °C. Following the treatment, 150 µL of the bacterial suspension (1.5 × 10^8^ CFU/mL) was incubated with 150 µL of PBS containing 0.125 mM H_2_O_2_ for 1 h. Next, 30 µL of 3-[4,5-dimethylthiazol-2-yl]-2,5 diphenyl tetrazolium bromide (MTT; Sigma-Aldrich) was added to determine the viability percentage. Azithromycin (Sigma-Aldrich) served as a positive control and an extract-free culture served as a growth control. Each assay was performed in triplicate and the percentage of mortality was calculated [30,49,50].

##### Inhibition of Pyocyanin Production Assay

CBRPA ATCC-35032 was cultured in *Pseudomonas* broth (PB; 10 g tryptone, 1.4 g MgCl_2_, 10 g KH_2_PO_4_, and 10 g C_6_H_5_Na_3_O_7_). Briefly, *P. aeruginosa* was grown on PB to match the 0.5 McFarland standard turbidity. This suspension was diluted 1:50 to prepare the working inoculum, and 1000 μL of this suspension was incubated with 1000 μL of PB containing the *n*-Hex plant extract at MIC/2 in 24-well plates. The cultures were incubated at 37 °C with shaking (120 rpm) for 48 h. The supernatant was collected by centrifugation at 13,000 rpm for 30 min. Pyocyanin production was visually observed as a blue-green color in the supernatant. Two hundred supernatants were transferred to a 96-well microplate to determine pyocyanin concentration spectrophotometrically at 690 nm. Azithromycin served as a positive control and untreated bacterial cultures were used as negative controls. The percentage of inhibition of pyocyanin production at each extract concentration was calculated. Each assay was conducted in triplicate and the mean ± standard deviation was calculated [51].

### 4.3. Cytotoxicity Assay

Vero cell viability (ATCC CCL-8) was assessed using the sulforhodamine B (SRB; (Sigma-Aldrich) assay. The cells were cultured in 96-well plates with DMEM and 10% fetal bovine serum until they reached 90% confluency. The medium was then replaced with 200 μL of fresh serum-free medium containing *n*-Hex plant extracts at serial dilutions ranging from 1000 to 31.25 μg/mL. Incubation was performed at 37 °C with 5% CO_2_ for 48 h, followed by the removal of the medium. Cells were fixed with trichloroacetic acid, stained with SRB, and solubilized. The absorbance was measured at 540 nm using a microplate reader. Docetaxel (Sigma-Aldrich) and untreated cells were used as positive and negative controls, respectively. The results are presented as CC_90_. All tests were performed in triplicate, and the CC_90_ values were calculated using GraphPad Prism ver. 5 software [52].

### 4.4. Chemometric Analysis Based on GC-MS

MetaboAnalyst V.6.0 (https://www.metaboanalyst.ca; accessed on 17 October 2024) was employed to perform a PLS-DA to correlate the chemical composition of the *n*-Hex plant extracts with their anti-infective activity. The phytoconstituents identified by GC–MS profiling (% peak area) were organized into an Excel spreadsheet to form matrix X. Matrix Y represented the anti-infective properties of the *n*-Hex plant extracts, categorized by activity levels. The R² and Q² values were calculated using a 5-fold cross-validation method to evaluate the model’s fit and predictive ability. VIP and correlation values from the PLS-DA analysis were used to identify compounds that significantly contributed to clustering and discrimination [53].

### 4.5. Statistical Analysis

R. software (v.4.3.2) was used for data analysis. The normality of the inhibition percentage data was assessed using the Shapiro–Wilk test. One-way analysis of variance (ANOVA) was performed to determine statistically significant differences between the data, followed by Tukey’s post hoc test. Results with *p*-values > 0.05 were deemed non-significant in all statistical analyses.

## 5. Conclusions

This study comprehensively investigated the effects of non-polar extracts of *B. potosina* and *T. paucidentata* on the growth, resistance mechanisms, and production of virulence factors in two priority antibiotic-resistant bacteria, CBRAB and CBRPA. The *n*-Hex liana extract of *B. potosina* demonstrated the most potent and broad-spectrum antibacterial properties against CBRAB and CBRPA strains. Notably, extracts with antibacterial activity do not necessarily affect the resistance mechanisms or virulence factors. The *n*-Hex bark extract from *T. paucidentata* exhibited low anti-biofilm activity, whereas its leaf extract displayed strong carbapenem-modulation activity against CBRAB but not against CBRPA. The *n*-Hex flower extract from *B. potosina* inhibited the virulence of *A. baumannii* by reducing catalase activity and suppressing pyocyanin production in *P. aeruginosa.* The application of PLS-DA to analyze the extracts resulted in the identification of biomarkers associated with anti-infective properties. This approach has proven effective in identifying potential biomarkers for the development of multi-target anti-infective phytodrug. This study enhances our understanding of the pharmacological properties and chemical compositions of these two Mayan medicinal plants. This highlights the importance of re-evaluating the traditional medicinal practices of indigenous communities and exploring the potential of plant species to combat the silent AMR pandemic.

## Figures and Tables

**Table 1:** Chemical profile of *n*-Hex extracts from various organs of *Bignonia potosina.*

Peak No.	Rt (min)	Compound Name	Molecular Formula	Molecular Weight	Peak Area (%)		
					BPLi	BPF	BPL
1	5.35	nitrocyclohexane	C_6_H_11_NO_2_	129	-	-	19.2
2	19.60	2-pentadecanone	C_15_H_30_O	226	-	2.6	-
3	20.84	tetradecanoic acid	C_14_H_28_O_2_	228	-	0.4	-
4	22.30	2-pentadecanone, 6,10,14-trimethyl-	C_18_H_36_O	268	1.1	0.1	-
5	23.07	2-heptadecanone	C_17_H_34_O	254	-	0.5	-
6	24.60	hexadecanoic acid	C_16_H_32_0_2_	256	12.4	23.9	-
7	27.50	hexadecanamide	C_16_H_33_NO	255	22.9	6.7	2.1
8	29.09	not identified			0.8	2.5	-
9	30.25	octadecanamide	C_18_H_37_NO	283	12.0	3.8	2.5
10	31.78	pentacosane	C_25_H_52_	352	2.5	8.2	-
11	32.97	not identified			3.1	1.6	-
12	34.17	octacosane	C_28_H_28_	394	3.1	12.3	-
13	34.69	1,4-benzenedicarboxylic acid, bis(2-ethylhexyl) ester	C_24_H_38_O	390	1.3	0.7	-
14	35.35	not identified			2.6	1.4	-
15	35.70	squalene	C_30_H_50_	410	-	-	42.8
16	36.18	α-tocospiro	C_29_H_50_O	462	1.0	-	-
17	36.58	nonacosane	C_29_H_60_	408	4.8	10.5	-
18	37.58	not identified			2.6	1.0	-
19	38.66	tetratriacontane	C_34_H_70_	478	7.9	10.4	14.2
20	39.69	not identified			2.2	-	0.65
21	40.12	stigmasterol	C_29_H_48_O	412	3.7	0.1	-
22	40.70	hexatriacontane	C_36_H_74_	507	10.0	4.2	7.0

min: minutes; Rt: retention time; BPLi: *n*-Hex liana extract; BPF: *n*-Hex flower extract; BPL: *n*-Hex leaf extract.

**Table 2:** Chemical profile of n-Hex extracts from various organs of *Thouinia paucidentata*.

Peak No.	Rt (min)	Compound Name	Molecular Formula	Molecular Weight	Peak Area (%)	
					TPB	TPL
1	5.35	nitrocyclohexane	C_6_H_11_NO_2_	129	8.1	-
2	18.27	β-elemene	C_15_H_24_	204	-	0.6
3	18.95	β-caryophyllene	C_15_H_24_	204	0.6	5.2
4	19.78	α-humulene	C_15_H_24_	204	-	2.3
5	19.95	9-epi-trans-caryophyllene	C_15_H_24_	204	-	1.3
6	20.48	β-copaene	C_15_H_24_	204	-	4.0
7	20.59	β-selinene	C_15_H_24_	204	-	0.7
8	21.52	cadina-1(10),4-diene	C_15_H_24_	204	-	1.3
9	22.82	cubebol	C_15_H_26_O	222	-	18.8
10	22.92	caryophyllene oxide	C_15_H_24_O	220	43.0	1.2
11	23.2	humulene epoxide II	C_15_H_24_O	220	1.3	-
12	23.5	bicyclo[7.2.0]undecan-3-ol	C_15_H_24_O	220	2.8	-
13	24.60	hexadecanoic acid	C_16_H_32_0_2_	256	2.8	-
14	26.39	oplopanone	C_15_H_26_O_2_	238	-	8.2
15	27.5	hexadecanamide	C_16_H_33_NO	255	7.1	-
16	28.08	not identified			-	3.9
17	28.72	kolavelool	C_20_H_34_O	290	23.8	1.6
18	29.86	cembrenol	C_20_H_34_O	290	-	5.4
19	30.24	not identified			3.7	-
19	30.76	cembrene A	C_20_H_32_	272	-	1.3
20	32.56	thunbergen	C_20_H_32_	272	-	23.3
21	42.95	not identified			-	1.17
22	44.74	squalene	C_30_H_50_	410	-	1.2
23	45.71	not identified			-	1.17
24	48.29	octadecane, 3-ethyl-5-(2-ethylbutyl)-	C_26_H_54_	366	-	0.8
25	48.69	α-tocopherol	C_29_H_50_O_2_	430	-	4.6
26	49.69	ethyl cholate	C_26_H_44_O_5_	436	-	0.6
27	50.07	stigmasterol	C_29_H_48_O	412	1.3	1.9
28	50.77	ç-Sitosterol	C_29_H_50_O	414	1.2	2.0
29	51.08	α-amyrin	C_30_H_50_O	426	-	2.3
30	51.62	lupeol	C_30_H_50_O	426	-	1.8

min: minutes; Rt: retention time; TPB: n-Hex bark extract; TPL: n-Hex leaf extract.

**Table 3:** Antibacterial activity of *n*-Hex extracts of *Bignonia potosina* and *Thouinia paucidentata* against *A. baumannii* strains.

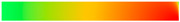 Most Active Less Active																
Part of Plants or Antibiotic		ID	MIC and MBC (μg/mL)													
			UIMY-ABA-													
			ATCC 1605 XDR CBR		16 MDR CBR		81 MDR CBR		7 XDR CBR		5 XDR CBR		205 XDR CBR		63 PDR CBR	
			MIC	MBC	MIC	MBC	MIC	MBC	MIC	MBC	MIC	MBC	MIC	MBC	MIC	MBC
*Bignonia potosina*	Liana	BPLi	31.25	250	15.7	15.7	31.25	250	31.25	250	31.25	250	15.7	250	31.25	250
	Flower	BPF	250	>1000	62.5	62.5	125	>1000	125	1000	125	1000	125	1000	250	>1000
	Leaf	BPL	>1000	–	>1000	–	500	1000	>1000	–	>1000	–	>1000	–	>1000	–
*Thouinia paucidentata*	Bark	TPB	>1000	–	1000	1000	500	>1000	>1000	–	125	>1000	500	500	1000	>1000
	Leaf	TPL	>1000	–	1000	1000	1000	>1000	>1000	–	>1000	–	>1000	–	1000	1000
Colistin		COL	1	–	2	–	1	–	2	–	1	–	2	–	4	–

ID: identity; MDR: multidrug-resistant; PDR: pandrug-resistant: XDR: extensively drug-resistant; CBR: Carbapenem-resistant; MIC: minimum inhibitory concentration; MBC: minimum bactericidal concentration; –: Not determined. The underlined style corresponds to bactericidal extract (MBC/MIC ratio ≤ 4). Heat map: increasing redness indicates lower activity, while increasing greenness denotes higher activity of the extract.

**Table 4:** Antibacterial activity of *n*-Hex extracts of *Bignonia potosina* and *Thouinia paucidentata* against *P. aeruginosa* strains.

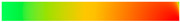 Most Active Less Active														
Part of Plants or Antibiotic		ID	MIC and MBC (μg/mL)											
			UIMY-PAE-											
			ATCC 19429 CBS		ATCC 27853 MDR CBR		ATCC 35032 MDR CBR		6 MDR CBR		79 MDR CBR		167 MDR CBR	
			MIC	MBC	MIC	MBC	MIC	MBC	MIC	MBC	MIC	MBC	MIC	MBC
*Bignonia potosina*	Liana	BPLi	500	1000	500	1000	250	500	1000	>1000	250	500	250	500
	Flower	BPF	>1000	–	>1000	–	>1000	–	>1000	–	1000	1000	>1000	–
	Leaf	BPL	>1000	–	>1000	–	>1000	–	>1000	–	>1000	–	>1000	–
*Thouinia paucidentata*	Bark	TPB	>1000	–	>1000	–	>1000	–	>1000	–	>1000	–	>1000	–
	Leaf	TPL	>1000	–	>1000	–	>1000	–	>1000	–	>1000	–	>1000	–
Amikacin		AMK	0.50	–	0.25	–	0.25	–	0.25	–	0.13	–	0.50	–

ID: identity; MDR: multidrug-resistant; PDR: pandrug-resistant: XDR: extensively drug-resistant; CBS: Carbapenem-susceptible; CBR: Carbapenem-resistant; MIC: minimum inhibitory concentration; –: Not determined. The underlined style corresponds to bactericidal extract (MBC/MIC ratio ≤ 4). Heat map: increasing redness indicates lower activity, while increasing greenness denotes higher activity of the extract.

**Table 5:** MIC of IMP in combination with extracts of *Bignonia potosina* or *Thouinia paucidentata* against CBRAB UIMY-ABA-5.

Extract	IMP Plus Extract	AMF
None	128	—
BPLi ^g^	16	8
BPLi ^h^	32	4
BPLi ^i^	128	1
BPF ^e^	64	2
BPF ^f^	64	2
BPF ^g^	128	1
BPL ^a^	8	16
BPL ^b^	32	4
BPL ^c^	64	2
TPB ^c^	32	4
TPB ^d^	32	4
TPB ^e^	128	1
TPL ^a^	0.0625	2048
TPL ^b^	16	8
TPL ^c^	128	1
PaβN ^j^	8	16

MIC: minimal inhibitory concentration; IMP: imipenem; AMF: antibiotic-modulating factor; PAβN: phenylalanine-arginine-β-naphthylamide. ^a^ 1000 µg/mL; ^b^ 500 µg/mL; ^c^ 250 µg/mL; ^d^ 125 µg/mL; ^e^ 62.5 µg/mL; ^f^ 31.25 µg/mL; ^g^ 7.8 µg/mL; ^h^ 3.9 µg/mL; ^i^ 1.95 µg/mL; ^j^ 50 µg/mL From *B. potosina*: BPLi: *n*-Hex liana extract; BPF: *n*-Hex flower extract; BPL: *n*-Hex leaf extract. From *T. paucidentata* TPB: *n*-Hex bark extract; TPL: *n*-Hex leaf extract.

**Table 6:** MIC of MEM in combination with extracts of *Bignonia potosina* or *Thouinia paucidentata* against CBRPA UIMY-PAE-167.

Extract or Compound	MEM Plus Extract	AMF
None	64	—
BPLi ^d^	16	4
BPLi ^e^	32	2
BPLi ^f^	64	1
BPF ^a^	32	2
BPF ^b^	32	2
BPF ^c^	64	1
BPL ^a^	32	2
BPL ^b^	32	2
BPL ^c^	64	1
TPB-1 ^a^	16	4
TPB-1 ^b^	32	2
TPB-1 ^c^	64	1
TPL-1 ^a^	32	2
TPL-1 ^b^	32	2
TPL-1 ^c^	64	1
PaβN ^g^	16	4

MIC: minimal inhibitory concentration; MEM: meropenem; AMF: antibiotic-modulating factor; PAβN: phenylalanine-arginine-β-naphthylamide. ^a^ 1000 µg/mL; ^b^ 500 µg/mL; ^c^ 250 µg/mL; ^d^ 125 µg/mL; ^e^ 62.5 µg/mL; ^f^ 31.25; ^g^ 50 µg/mL. From *B. potosina*: BPLi: *n*-Hex liana extract; BPF: *n*-Hex flower extract; BPL: *n*-Hex leaf extract. From *T. paucidentata* TPB: *n*-Hex bark extract; TPL: *n*-Hex leaf extract.

**Table 7:** Summary of putative biomarkers identified by PLS-DA (VIP > 1) from *n*-Hex *Bignonia potosina* and *Thouinia paucidentata* extracts associated with anti-infective activity against CBRAB and CBRPA strains.

Compound Name	Class	Associated Activity	VIP	*r* Value ^a^
hexadecanoic acid	fatty acid	anti-growth	2.37	0.45
		anti-virulence	3.46	0.49
hexadecanamide	fatty acid amide	anti-growth	2.75	0.36
octadecanamide	fatty acid amide	anti-growth	1.47	0.24
thunbergen	diterpene	antibiotic-modulation	2.86	0.37
cubebol	sesquiterpene	antibiotic-modulation	2.10	0.28
octacosane	higher alkane	anti-virulence	2.50	0.30

^a^ r value determined for component 1.

**Table 8:** Cytotoxicity of *Bignonia potosina* and *Thouinia paucidentata n*-Hex extracts on Vero cells.

	BPLi	BPF	BPL	TPB	TPL	Docetaxel
CC_90_	235.2 ± 4.5 ^d^	624.4 ± 3.1 ^a^	>1000	292.7 ± 3.3 ^c^	398.1 ± 3.7 ^b^	7.8 ± 0.7 ^e^
SI ^a^	15.0–7.5	10–2.5	-	2.3–<0.3	0.4–<0.4	-
SI ^b^	0.9–<0.2	0.6–<0.6	-	<0.3	<0.4	-

CC90 in µg/mL. Average CC90 values ± SD with the same letter showed non-significant differences in the post hoc Tukey test (*p* < 0.05). Sia = CC90/MIC values anti-growth against CBRAB strains. Sib = CC90/MIC anti-growth against CBRPA strains. From *B. potosina*: BPLi: *n*-Hex liana extract; BPF: *n*-Hex flower extract; BPL: *n*-Hex leaf extract. From *T. paucidentata* TPB: *n*-Hex bark extract; TPL: *n*-Hex leaf extract.

**Table 9:** Phenotypic profile of *Acinetobacter baumannii* strains.

Identity	Source/Biological Sample	Drug-Resistant Phenotype	Drug Resistant to:
1605	ATCC	XDR, CBR	AMP, CAZ, CFZ, CIP, CRO, CTX, ETP, FEP, FOS, FOX, GEN, IPM, LVX, MEM, SAM, SXT, TZP
UIMY-ABA-16	CI from urine	MDR, CBS	AMK, CAZ, CIP, CRO, CTX, FEP, GEN, LVX, SXT, TET, TOB
UIMY-ABA-81	CI from pleural liquid	MDR, CBR	AMK, CAZ, CIP, CRO, CTX, FEP, GEN, LVX, MEM, SXT, TOB
UIMY-ABA-7	CI from bronchial liquid	XDR, CBR	AMK, CAZ, CIP, CRO, CTX, FEP, GEN, LVX, MEM, SAM, SXT, TET, TOB
UIMY-ABA-5	CI from urine	XDR, CBR	AMK, CAZ, CIP, CRO, CTX, FEP, GEN, IMP, LVX, MEM, SAM, SXT, TET, TOB
UIMY-ABA-205	CI from blood	XDR, CBR	AMK, CAZ, CIP, FEP, GEN, IMP, LVX, MEM, SXT, TZP
UIMY-ABA-63	CI from bronchial liquid	PDR, CBR	AMK, CAZ, CIP, COL, CRO, CTX, FEP, LVX, MEM, SAM, SXT, TET, TOB

Clinical isolate: CI; CBS: Carbapenem-susceptible; CBR: Carbapenem-resistant; MDR: Multidrug-resistant; XDR: extensively drug-resistant; PDR: pandrug-resistant; AMK: Amikacin; AMP: Ampicillin; CAZ: Ceftazidime; CFZ: Cefazolin; CIP: Ciprofloxacin; COL: Colistin; CRO: Ceftriaxone; CTX: Cefotaxime; ETP: Ertapenem; FEP: Cefepime; FOS: Fosfomycin; FOX: Cefoxitin; GEN: Gentamycin; IPM: Imipenem; LVX: Levofloxacin; MEM: Meropenem; SAM: Ampicillin/Sulbactam; SXT: Trimethoprim/Sulfamethoxazole; TET: Tetracycline; TOB: Tobramycin; TZP: Piperacillin/Tazobactam.

**Table 10:** Phenotypic profile of *Pseudomonas aeruginosa* strains.

Identity	Source/Biological Sample	Drug Resistant Phenotype	Drug Resistant to:
19429	ATCC	CBS	-
27853	ATCC	MDR, CBR	AMP, CFZ, CRO, ETP, FOX, SAM, SXT, TGC
35032	ATCC	MDR, CBR	AMP, CFZ, CRO, CTX, ETP, FOX, SAM, SXT, TGC
UIMY-PAE-6	CI from bronchial liquid	MDR, CBR	CAZ, CIP, FEP, IPM, LVX, TIC
UIMY-PAE-79	CI from bronchial liquid	MDR, CBR	CIP, FEP, GEN, IPM, LVX, MEM, TZP
UIMY-PAE-167	CI from bronchial liquid	MDR, CBR	CAZ, CIP, FEP, GEN, IPM, LVX, TIC

Clinical isolate: CI; CBS: Carbapenem-susceptible; CBR: Carbapenem-resistant; MDR: Multidrug-resistant; AMP: Ampicillin; CAZ: Ceftazidime; CFZ: Cefazolin; CIP: Ciprofloxacin; CRO: Ceftriaxone; CTX: Cefotaxime; ETP: Ertapenem; FEP: Cefepime; FOX: Cefoxitin; GEN: Gentamycin; IPM: Imipenem; LVX: Levofloxacin; MEM: Meropenem; SAM: Ampicillin/Sulbactam; SXT: Trimethoprim/Sulfamethoxazole; TZP: Piperacillin/Tazobactam; TGC: Tigecycline; TIC: Ticarcillin/Clavulanic acid.

## Data Availability

Data are contained within the article.

## Supplementary Materials

The following supporting information can be downloaded at: https://www.mdpi.com/article/10.3390/plants13243498/s1, Table S1. Phytoconstituents identified by PLS-DA (VIP > 1) from *n*-Hex *Bignonia potosina* and *Thouinia paucidentata* extracts associated with anti-infective. Figure S1. Loading plots from PLS-DA model for activity on bacterial growth with all compounds. Figure S2. Loadings plot from PLS-DA model for antibiotic-modulation activity with all compounds. Figure S3. Loadings plot from PLS-DA model for antivirulence activity with all compounds.
