# Supplementary material for: Mayan Medicinal Plants Bignonia potosina and Thouinia paucidentata Demonstrate Anti-Infective Properties Against the Priority Antibiotic-Resistant Bacteria Acinetobacter baumannii and Pseudomonas aeruginosa
Source: Plants (Basel). 2024 Dec 14;13(24):3498. doi: 10.3390/plants13243498 (PMC11677589; doi:10.3390/plants13243498)
Supplement: Supplementary file 1 [file plants-13-03498-s001.zip › plants-3370569-supplementary.pdf]

Supplementary Materials

Table S1. Phytoconstituents identified by PLS-DA (VIP >1) from *n*-Hex *Bignonia potosina* and *Thouinia paucidentata* extracts associated with anti-infective.

Structure	Compound name	Class	Associated activity
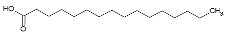	hexadecanoic acid	fatty acid	anti-growth<br>anti-virulence
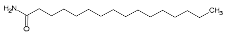	hexadecanamide	fatty acid amide	anti-growth
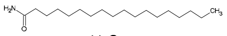	octadecanamide	fatty acid amide	anti-growth
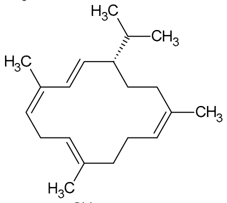	thunbergen	diterpene	antibiotic-modulation
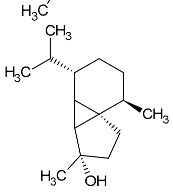	cubebol	sesquiterpene	antibiotic-modulation
	octacosane	higher alkane	anti-virulence

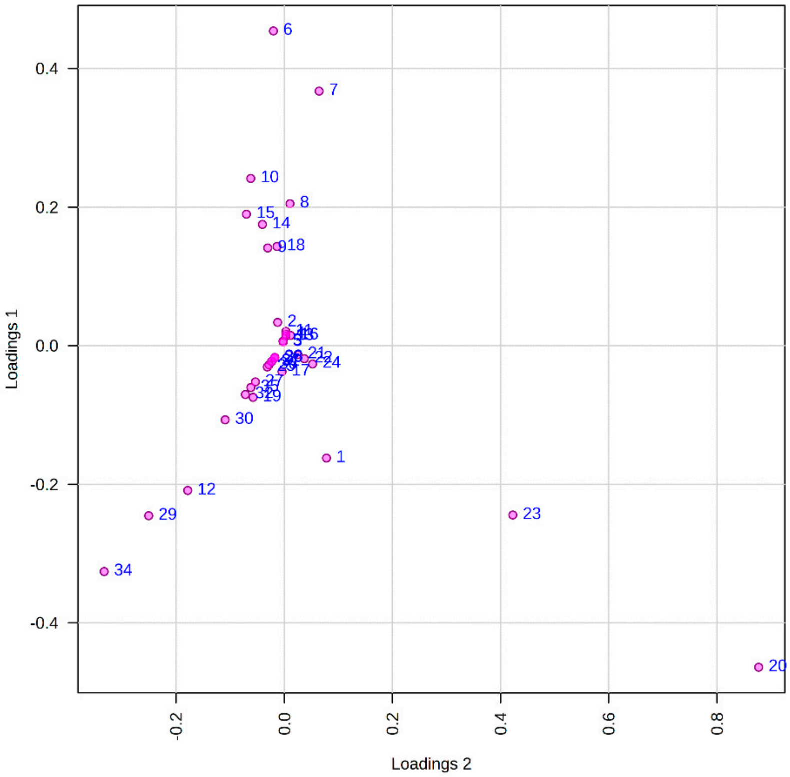

Figure S1. Loading plots from PLS-DA model for activity on bacterial growth with all compounds. 1: Nitrocyclohexane; 2: 2-pentadecanone; 3: tetradecanoic acid; 4: 2-pentadecanone, 6,10,14-trimethyl-; 5: 2-heptadecanone; 6: hexadecanoic acid; 7: hexadecanamide; 8: octadecanamide; 9: pentacosane; 10:

octacosane; 11:1,4-benzenedicarboxylic acid, bis(2-ethylhexyl) ester; 12: squalene; 13: alfa-tocospiro; 14: nonacosane; 15: tetratriacontane; 16: stigmasterol; 17: sitosterol; 18: hexatriacontane; 19: beta-caryophyllene; 20: caryophyllene oxide; 21: humulene epoxide II; 22: resorcylic acid; 23: kolavelool; 24: kolavenol; 25: alfa-humulene; 26: 9-epi-trans-caryophyllene; 27: beta-copaene; 28: cadina-1(10),4-diene; 29: cubebol ; 30: oplopanone; 31: isospathulenol; 32: cembrenol; 33: cembrene A; 34: thunbergen; 35: alfa-tocoferol; 36: alfa-amyrin; 37: lupeol.

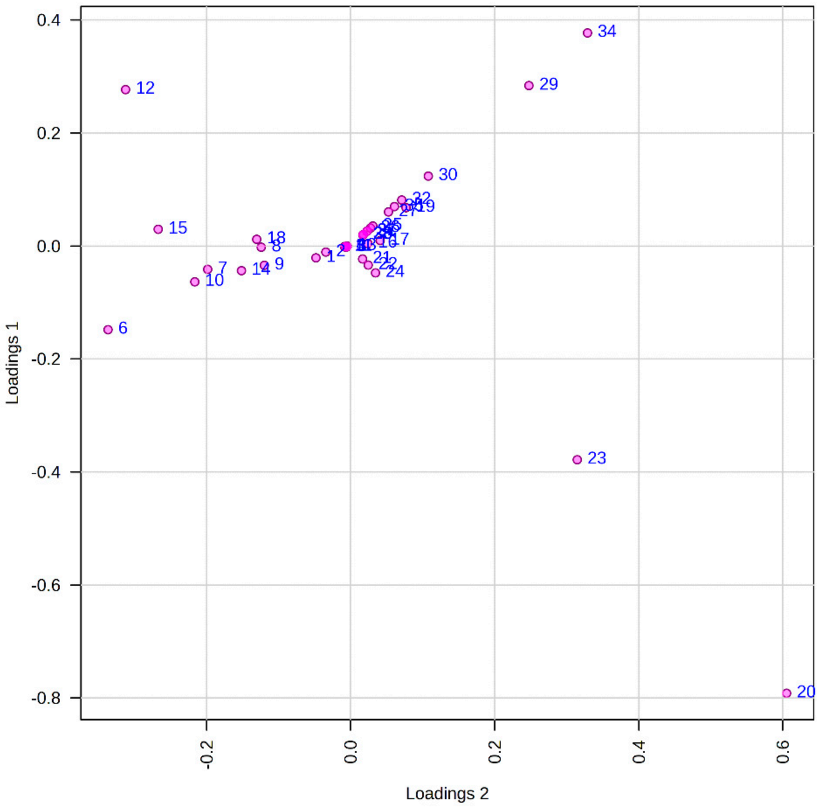

Figure S2. Loadings plot from PLS-DA model for antibiotic-modulation activity with all compounds. 1: Nitrocyclohexane; 2: 2-pentadecanone; 3: tetradecanoic acid; 4: 2-pentadecanone, 6,10,14-trimethyl-; 5: 2-heptadecanone; 6: hexadecanoic acid; 7: hexadecanamide; 8: octadecanamide; 9: pentacosane; 10: octacosane; 11:1,4-benzenedicarboxylic acid, bis(2-ethylhexyl) ester; 12: squalene; 13: alfa-tocospiro; 14: nonacosane; 15: tetratriacontane; 16: stigmasterol; 17: sitosterol; 18: hexatriacontane; 19: beta-caryophyllene; 20: caryophyllene oxide; 21: humulene epoxide II; 22: resorcylic acid; 23: kolavelool; 24: kolavenol; 25: alfa-humulene; 26: 9-epi-trans-caryophyllene; 27: beta-copaene; 28: cadina-1(10),4-diene; 29: cubebol ; 30: oplopanone; 31: isospathulenol; 32: cembrenol; 33: cembrene A; 34: thunbergen; 35: alfa-tocoferol; 36: alfa-amyrin; 37: lupeol.

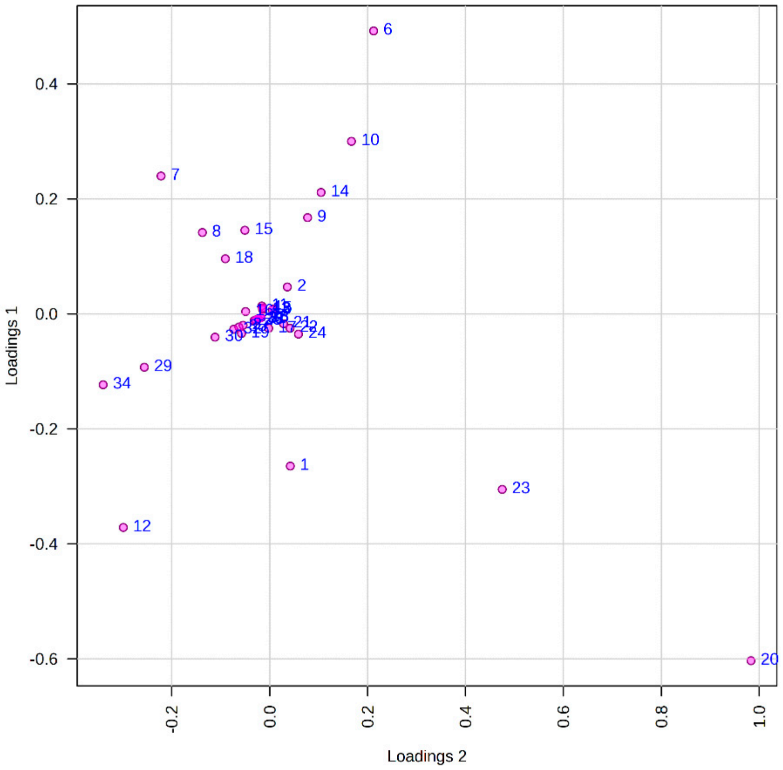

Figure S3. Loadings plot from PLS-DA model for antivirulence activity with all compounds. 1: Nitrocyclohexane; 2: 2-pentadecanone; 3: tetradecanoic acid; 4: 2-pentadecanone, 6,10,14-trimethyl-; 5: 2-heptadecanone; 6: hexadecanoic acid; 7: hexadecanamide; 8: octadecanamide; 9: pentacosane; 10: octacosane; 11:1,4-benzenedicarboxylic acid, bis(2-ethylhexyl) ester; 12: squalene; 13: alfa-tocospiro; 14: nonacosane; 15: tetratriacontane; 16: stigmasterol; 17: sitosterol; 18: hexatriacontane; 19: beta-caryophyllene; 20: caryophyllene oxide; 21: humulene epoxide II; 22: resorcylic acid; 23: kolavelool; 24: kolavenol; 25: alfa-humulene; 26: 9-epi-trans-caryophyllene; 27: beta-copaene; 28: cadina-1(10),4-diene; 29: cubebol ; 30: oplopanone; 31: isospathulenol; 32: cembrenol; 33: cembrene A; 34: thunbergen; 35: alfa-tocoferol; 36: alfa-amyrin; 37: lupeol.
